# The stability and robustness analysis of SNG and PNG supply

**DOI:** 10.1016/j.mex.2020.100802

**Published:** 2020-01-23

**Authors:** Jianhua Zhu, Zhuping Gong, Yuming Guo, Qing Wang

**Affiliations:** a School of Business Administration, South China University of Technology, Guangzhou, 510641, China; b Information: Mechanical and Electrical Engineering College, Jinggangshan University, Jian, 343009, China; c School of Mathematics, South China University of Technology, Guangzhou, 510641, China

**Keywords:** The stability and robustness analysis of SNG and PNG supply, Dynamic analysis, SNG and PNG, Stability, Perturbation, Robustness

## Abstract

Current researches on dynamic analysis of energy supply mainly (1) Chaotic control of supply process. (2) Resource synchronization in supply process. (3) Dynamic behavior of energy supply under the influence of parameter perturbation. However, in above researches, the disturbance type is not described and classified. In addition, none of the three approaches addresses the stability of the energy supply. In order to improve the above methods, in this paper, the stability analysis method of energy supply process is firstly proposed, this method can judge whether the deterministic energy supply system is stable at different times. However, the disadvantage of this method is that it is suitable for non-chaotic deterministic systems, but internal and external disturbances of the energy supply will make the supply system unstable and fall into chaos. So we classify the types of disturbances, give robustness of the system under different disturbances, and help company design energy supply system and select the system parameters with good robustness to analyze stability of energy supply process.

• The internal and external disturbances of the system are classified and discussed.
• Proposed the concept of robustness of energy supply system, and give the method to judge the robustness of the system.

## Graphical abstract

**Figure:**
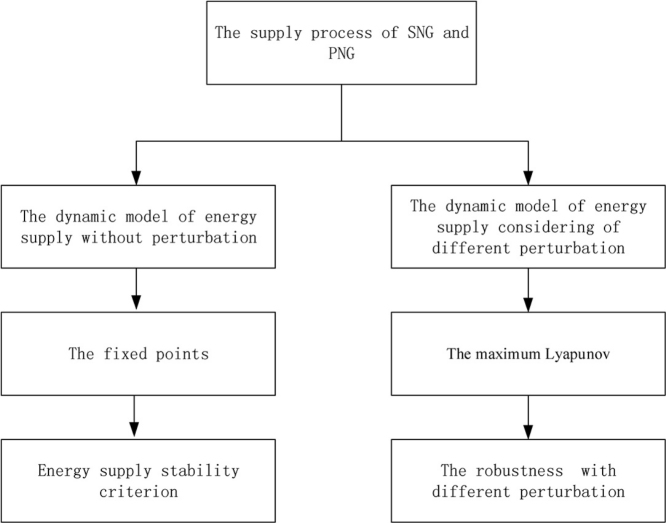

**Specification Table**

**Table:** 

Subject Area:	Energy
More specific subject area:	Energy supply
Method name:	The stability and robustness analysis of SNG and PNG supply
Name and reference of original method:	Time delayed feedback control [Sun M, Tian L-X, Xu J. Time delayed feedback control of the Energy Resource Chaotic System. International Journal of Nonlinear Science 2006; 1 (3):172-177]. Hopf Bifurcation Analysis [Sun M, Tian L-X, Yin J. Hopf Bifurcation Analysis of the Energy Resource Chaotic System. International Journal of Nonlinear Science 2006; 1 (1):pp.49-53]. Dynamics and adaptive synchronization [Sun M, Tian L-X, Fu Y, Qian W. Dynamics and adaptive synchronization of the energy resource system. Chaos, solitons and Fractals 2007; 31: 879-888]. Parametric perturbations and its hyper chaos control [Sun M, Tian L-X, Zeng C-Y. The energy resources system with parametric perturbations and its hyper chaos control. Nonlinear Analysis: Real World Applications 2009; 10: 2620-2626].
Resource availability:	Synthetic natural gas (SNG) and pipeline natural gas (PNG)

## Method details

Due to the shortage of natural gas energy, natural gas composed of SNG and PNG is typically supplied to satisfy customers’ demand in china. However, problems often arise with the supply system, resulting in an unstable supply of natural gas and causing great inconvenience to people's lives. Therefore, understanding how to maintain the robustness of the natural gas supply system and the stability of this supply in an uncertain environment is key to solving the above problems. Thus, the current paper focuses on the dynamic analysis of SNG and PNG supply from the perspective of stability and robustness.

• A new natural gas supply system model composed of SNG and PNG is constructed, and the dynamic behavior of the system supply process is analyzed.
• Lyapunov stability theory is used to analyze the stability of the dynamic supply process of natural gas supply system.
• The Bifurcation theory is used to analyze the robustness of the natural gas supply system in an uncertain environment.

### Modelling approach

A diverse variety of modelling techniques have been developed to solve specific problems focusing on the stability and reliability of energy supply in an uncertain environment, with some classical models that have made outstanding contributions.

In order to analyze the vulnerability and reliability of the natural gas supply system in a random environment, many approaches have been developed. Stochastic simulation methods such as the Monte Carlo-based method [1,2], Markov Process-based method [3], and the probability-based dynamic modelling method [4], among others [[5], [6], [7], [8], [9]], have been widely used to model uncertain natural gas supply systems. For example, a sequential Monte Carlo model was developed in Ref [8]. to analyze disruptions in the gas transmission network. In Ref. [9], a capacity network stochastic model is developed, based on Markov modelling and graph theory. The model is embedded in an optimization algorithm to compute the capacities of the pipeline network under different scenarios and analyze the consequences of failures of units in the system. Ref [10]. considered both gas supply capacity and market demand uncertainties, and integrated the two calculation results into a Monte Carlo simulation. In addition, in Ref. [11], a transient gas simulation model was used for a supply security analysis.

In addition, graph theories to examine vulnerability and reliability, such as the topology-based method [12,13], stream-based method [14,15] and hybrid method [16,17] have been widely used. The Structural-Function modelling method based on graph theory and system engineering has often been used [18]. For example, in Refs. [19,20], a universal method based on a topological structure analysis and hydraulic calculation was used to quantify the gas supply reliability of a long-distance gas pipeline network system. In Ref. [21], the analysis of demand robustness evaluates the capacities of demand sites to withstand the strains imposed on the pipeline network system and further explains the differences in capacities, from a graph theory perspective. Sukharev's [22] graph theory and stochastic process theory have also been used, and multiple failures in the gas supply assessment considered.

In order to analyze the operation, function, capacity and limitations of a gas supply system, models such as GEMFLOW [23] and MC-GENGERCIS [24] have been constructed. For example, the latter was proposed to help decision makers respond suitably to supply crisis situations [24], specifically in order to assess the robustness of the EU transnational gas transmission system during normal and special operating conditions. Due to ignorance of the time evolution of the gas crisis in the MC-GENERCIS model, a new tool called GEMFLOW was introduced in Ref. [23]. by the same authors to assess possible outcomes of a supply disruption before e and minimise losses during e an emergency by determining the optimal distribution of flows. By employing the GEMFLOW model, the progress made in strengthening Europe's gas supply security was considered more economical than MC-GENGERCIS model for evaluating the overall security of China's natural gas supply system.

Most existing models study the randomness, stability and safety of the natural gas supply process. However, the dynamic behavior analysis of a natural gas supply system is always ignored in previous gas supply. In order to make up for this lack, in Ref. [32], the dynamic model of energy logistics demand based on a dynamic energy logistics system was established. By analyzing the dynamic behavior of the model, the authors found that the values of the system parameters determined the dynamic behavior of the system. According to the stability of the equilibrium point, the range of system parameters variation was given. The insight that nonlinear dynamics theory is an effective approach with which to analyze the dynamic behavior of a natural gas supply system was generated by the above research.

Following the idea of nonlinear dynamics, a large number of studies were subsequently consulted for the current paper. While there is no nonlinear dynamics research on how SNG and PNG supply meet customers' demand, Sun's studies of energy provide a series of clues. In Ref. [25], Sun established a three-dimensional energy resource, chaotic DEN system, and investigated its control performance using the time delayed feedback control method. In Ref. [26], the authors studied Hopf Bifurcation on the basis of previous studies. In Ref. [27], they proposed a new threedimensional energy resource chaotic system and addressed the synchronization problem of a two-resource system. In Ref. [28], the authors analyzed the dynamic behavior of the three-dimensional energy system with parametric perturbations. In Ref. [29], they analyzed the three-dimensional energy resources demand-supply system and undertook empirical research based on a non-linear approach. In Ref. [30], the authors reported on a new fourdimensional energy resource chaotic system, which was obtained by adding a new variable to the three-dimensional, demand-supply energy resource system established for two regions of China. In Ref. [31], the four dimensional energy supply-demand systems were proved to be a nonlinear complex system.

The studies of Refs. [[25], [26], [27], [28], [29], [30], [31], [32]] show that a nonlinear dynamic system and chaos equation may be a suitable way to solve the problem of SNG and PNG synchronized supply. Thus, the current paper focuses on the dynamic analysis of SNG and PNG supply from the perspective of stability and robustness. On one hand, the stability of the natural gas supply is analyzed using the stability theory of nonlinear dynamics. On the other hand, due to the natural gas supply process being prone to internal disturbance and external disturbance, bifurcation theory is used to analyze the impact of disturbance on the natural gas supply, ultimately providing the conditions for the robust operation of the natural gas supply system. Research on SNG and PNG supply reliability and robustness plays an important role in today's energy industry. This includes ensuring the safety of the gas supply, proposing measures for improving gas supply reliability and robustness, and improving the economic and social benefits of gas suppliers.

#### Nonlinear and dynamic

The natural gas supply system consisting of PNG and SNG is a complex system. In the natural gas supply process, SNG and PNG achieve the supply of natural gas through a nonlinear interaction. The interaction between SNG and PNG is mainly reflected in the dynamic of competition and cooperation. The competitive relationship is reflected in the fact that one source of energy will inhibit the output of the other, while the cooperative relationship is reflected in the fact that the two gases work together to fulfill customers’ demand for natural gas. Based on the Population Competition model [32], the natural gas supply system consisting of PNG and SNG was modeled and analyzed. Then, Lyapunov’s stability theory and Bifurcation theory are applied to the problem under analysis by using nonlinear dynamics method [[25], [26], [27], [28]]. Finally, a dynamic analysis of the supply process of the natural gas supply system was carried out.

#### Modelling assumptions

All energy supply system models are based on a set of assumptions and necessary simplifications, and the gas supply system established in this paper is no exception. As a two-dimensional nonlinear dynamic system, the natural gas dynamic system established here describes the dynamic behavior relationship between PNG and SNG in the process of supplying natural gas. In the establishment and analysis of the model, the following conditions are always assumed to be true:

• The supply of natural gas always meets users’ demand.
• The supply of natural gas is a self-organizing process without human intervention.
• In the process of supplying natural gas, the disturbance to SNG is relatively stable, while the disturbance to PNG is relatively unstable but still satisfies a certain rule.

Firstly, as researched in Ref. [33], the competitive relationship between SNG and PNG can be expressed by the variance of SNG and PNG, respectively. The equations are as follows:

(1) $$\left\{\begin{array}{c} \frac{dy_{s}1}{dt}=-a_{1}y_{p_{1}}\\ \frac{dy_{p}1}{dt}=-b_{1}y_{s_{1}} \end{array}\right)$$

where *y_s_*, *y_p_* represent SNG and PNG, respectively; -*a_1_*<0, -*b_1_*<0 are inhibitory factors; -*a_1_* indicates that PNG will inhibit the natural gas supply emanating from SNG; -*b_1_* indicates that SNG will inhibit the natural gas supply emanating from PNG; *a_1_* and *b_1_* are influenced by the rate of gas conversion, gas supply speed, the price of gas, and so on.

Secondly, the cooperative relationship between SNG and PNG in the gas supply system was analyzed. It was found that the demand for natural gas drives the supply of SNG and PNG until the gas supply reaches its threshold. However, once the latter occurs, the driving effect disappears. The following model was established to express this discovery:

(2) $$\left\{\begin{array}{c} \frac{dy_{s_{2}}}{dt}=a_{2}\left(X_{g}-y_{p}\right)\left[N_{s}-\left(X_{g}-y_{p}\right)\right]\\ \frac{dy_{p_{2}}}{dt}=b_{2}\left(X_{g}-y_{s}\right)\left[N_{p}-\left(X_{g}-y_{s}\right)\right] \end{array}\right)$$

where *a_2_* represents the influence of natural gas demand surplus on SNG supply change rate within the range of maximum load surplus of SNG.*b_2_* represents the influence of natural gas demand surplus on PNG supply change rate within the range of maximum load surplus of PNG. It should be noted that *a_2_* and *b_2_* are constants which are influenced by factors such as the cost of producing gas, the governance cost caused by the respective SNG and PNG pollution, and so on*. N_s_* represents the threshold of SNG and *N_p_* the threshold of PNG. *X_g_* denotes the demand for natural gas. Generally speaking, consumers' demand for natural gas is stochastic, thus *X_g_* is a variable. In order to eliminate the uncertain effects of natural gas demand, the equation *X_g_*=*y_s_*+*y_p_* was constructed, meaning that Eq. (2) could be rewritten as follows:

(3) $$\left\{\begin{array}{c} \frac{dy_{s_{2}}}{dt}=a_{2}(N_{s}-y_{s_{2}})y_{s_{2}},\\ \frac{dy_{p_{2}}}{dt}=b_{2}(N_{p}-y_{p_{2}})y_{p_{2}}. \end{array}\right)$$

Finally, by combining the competitive and cooperative relationships between SNG and PNG in the gas supply system, the dynamic model of the simultaneous supply of gas by PNG and SNG was established. This can be expressed as follows:

(4) $$\left\{\begin{array}{c} \frac{dy_{s}}{dt}=-a_{1}y_{p}+a_{2}(N_{s}-y_{s})y_{s},\\ \frac{dy_{p}}{dt}=-b_{1}y_{s}+b_{2}(N_{p}-y_{p})y_{p}. \end{array}\right)$$

### Fixed points analysis

In order to reveal the change law of these two gases without manual intervention, the dynamic characteristics of the presented model (Eq. (4)) were analyzed by Ref. [33].

It is well known that stability is a very important index for any system. The state of a system will not change when the system is near the stable fixed points. Studying the stability of the fixed points can enable the judgment of whether the gas supply is in a stable state. For this purpose, it was first necessary to locate the fixed points of Eq. (4). Thus, Eq. (4) was written in the form of equation Eq. (5):

(5) $$\left\{\begin{array}{c} f(y_{s},y_{p})=-a_{1}y_{p}+a_{2}(N_{s}-y_{s})y_{s}\\ g(y_{s},y_{p})=-b_{1}y_{s}+b_{2}(N_{p}-y_{p})y_{p} \end{array}\right)$$

In order to obtain the fixed points of the gas supply system, the formulation *f*(*y_s_*,*y_p_*)=*g(y_s_,y_p_)* = 0 was made and Eq. (5) calculated. The solutions to Eq. (5) were first supposed to be *P_1_(y_s_^(1)^,y_p_^(1)^)*, *P_2_(y_s_^(2)^,y_p_^(2)^)*, *P_3_(y_s_^(3)^,y_p_^(3)^)* and *P_4_(0,0)*,

(6) $$\begin{array}{ll} \text{where} & y_{s}^{\left(1\right)}=\sqrt[{3}]{-\frac{q}{2}+\sqrt{{\left(\frac{q}{2}\right)}^{2}+{\left(\frac{p}{3}\right)}^{3}}}+\sqrt[{3}]{-\frac{q}{2}-\sqrt{{\left(\frac{q}{2}\right)}^{2}+{\left(\frac{p}{3}\right)}^{3}}}+\frac{2}{3}N_{s},y_{p}^{\left(1\right)}=\frac{a_{2}}{a_{1}}y_{s}^{\left(1\right)}\left(N_{s}-y_{s}^{\left(1\right)}\right)\\ & y_{s}^{\left(2\right)}=\omega \sqrt[{3}]{-\frac{q}{2}+\sqrt{{\left(\frac{q}{2}\right)}^{2}+{\left(\frac{p}{3}\right)}^{3}}}+\omega^{2}\sqrt[{3}]{-\frac{q}{2}-\sqrt{{\left(\frac{q}{2}\right)}^{2}+{\left(\frac{p}{3}\right)}^{3}}}+\frac{2}{3}N_{s},y_{p}^{\left(2\right)}=\frac{a_{2}}{a_{1}}y_{s}^{\left(2\right)}\left(N_{s}-y_{s}^{\left(2\right)}\right)\\ & y_{s}^{\left(3\right)}=\omega^{2}\sqrt[{3}]{-\frac{q}{2}+\sqrt{{\left(\frac{q}{2}\right)}^{2}+{\left(\frac{p}{3}\right)}^{3}}}+\omega \sqrt[{3}]{-\frac{q}{2}-\sqrt{{\left(\frac{q}{2}\right)}^{2}+{\left(\frac{p}{3}\right)}^{3}}}+\frac{2}{3}N_{s},y_{p}^{\left(3\right)}=\frac{a_{2}}{a_{1}}y_{s}^{\left(3\right)}\left(N_{s}-y_{s}^{\left(3\right)}\right) \end{array}$$

where

$$\begin{array}{l} \omega =\frac{-1+\sqrt{3}i}{2}\\ p=\frac{\left(3\frac{a_{1}}{a_{2}}N_{m}-N_{c}^{2}\right)}{3},q=\frac{27\left[{\left(\frac{a_{1}}{a_{2}}\right)}^{2}\frac{b_{1}}{b_{2}}-\frac{a_{1}}{a_{2}}N_{m}N_{c}\right]+\left(\frac{a_{1}}{a_{2}}N_{c}N_{m}+2N_{c}^{3}\right)}{27} \end{array}$$

The relationships among the first three points are:

(7) $$\left\{\begin{array}{c} {\text{y}}_{\text{s}}^{\left(1\right)}\text{ +}{\text{y}}_{\text{s}}^{\left(2\right)}+{\text{y}}_{\text{s}}^{\left(3\right)}=2{\text{N}}_{\text{s}}\\ {\text{y}}_{\text{s}}^{\left(1\right)}{\text{y}}_{\text{s}}^{\left(2\right)}{\text{y}}_{\text{s}}^{\left(3\right)}=({\text{a}}_{1}\text{/}{\text{a}}_{2})[({\text{a}}_{1}\text{/}{\text{a}}_{2})({\text{b}}_{1}\text{/}{\text{b}}_{2})-{\text{N}}_{\text{p}}{\text{N}}_{\text{s}}]\\ \text{1/}{\text{y}}_{\text{s}}^{\left(1\right)}\text{+1/}{\text{y}}_{\text{s}}^{\left(2\right)}\text{+1/}{\text{y}}_{\text{s}}^{\left(3\right)}=[({\text{a}}_{1}\text{/}{\text{a}}_{2}){\text{N}}_{\text{p}}+{\text{N}}_{\text{s}}^{2}]\text{/}[({\text{a}}_{1}\text{/}{\text{a}}_{2})({\text{N}}_{\text{s}}{\text{N}}_{\text{p}}-({\text{a}}_{1}\text{/}{\text{a}}_{2})({\text{b}}_{1}\text{/}{\text{b}}_{2}))] \end{array}\right)$$

A local analysis method incorporating the Jacobian matrix was then applied to estimate the stability of fixed points; namely, if *P_i_* satisfies the condition of det(*J*)>0 and tr(*J*)<0, then *p_i_* is stable.

(8) $$\text{where}\;J=\left[\begin{array}{cc} \frac{\partial f}{\partial y_{s}}(P_{i}) & \frac{\partial f}{\partial y_{p}}(P_{i})\\ \frac{\partial g}{\partial y_{s}}(P_{i}) & \frac{\partial g}{\partial y_{p}}(P_{i}) \end{array}\right]$$

In order to reduce computational complexity, it was supposed that:

$$\Delta ={\left(\frac{q}{2}\right)}^{2}+{\left(\frac{p}{3}\right)}^{3},m=-f_{y_{c}}^{'}(P_{i})-g_{y_{m}}^{'}(P_{i}),n=f_{y_{c}}^{'}(P_{i})g_{y_{m}}^{'}(P_{i})-f_{y_{m}}^{'}(P_{i})g_{y_{c}}^{'}(P_{i})$$

Furthermore, *m* and *n* could be expressed as follows:

$$\begin{array}{c} n=-4\frac{a_{2}^{2}b_{2}}{a_{1}}y_{s}^{3}+6\frac{a_{2}^{2}b_{2}}{a_{1}}N_{s}y_{s}^{2}-2a_{2}b_{2}\left(N_{p}+\frac{a_{2}N_{s}}{a_{1}}\right)y_{s}+a_{2}b_{2}N_{s}N_{p}-a_{1}b_{1}\\ m=-\frac{2a_{2}b_{2}}{a_{1}}y_{s}^{2}+\left(2a_{2}+\frac{2a_{2}b_{2}}{a_{1}}N_{s}\right)y_{s}-\left(a_{2}N_{s}+b_{2}N_{p}\right) \end{array}$$

This yielded the understanding that det(*J*)>0 and tr(*J*)<0 are equal to *m>0* and *n>0.* That is to say, if fixed point *p_i_* is stable, then *m>0* and *n>0*.

Next, the above criteria (*m>0* and *n>0)* were used to yield the condition that the fixed point *p_i_* is a stable point.

Theorem 1 If Δ>0, there are three nonzero fixed points in the natural gas supply system, where *(y_s_^(1)^,y_p_^(1)^)* is located in the real number field, and *(y_s_^(2)^,y_p_^(2)^)* and *(y_s_^(3)^,y_p_^(3)^)* are located in the complex number field. As for the fixed point *(y_s_^(1)^, y_p_^(1)^)*, its stable condition can be expressed as follows:

$$y_{s}\in \left[\frac{1}{2}\left(\frac{a_{1}}{b_{1}}+N_{s}-\sqrt{{\left(\frac{a_{1}}{b_{1}}\right)}^{2}+N_{s}^{2}-2\frac{a_{1}}{a_{2}}N_{p}}\right),-2\sqrt[{3}]{{\left(\frac{a_{1}}{a_{2}}\right)}^{2}\frac{b_{1}}{b_{2}}}\right]$$

Namely, the parameters should satisfy the following conditions:

$$(1){\left(\frac{a_{1}}{b_{1}}\right)}^{2}+N_{s}^{2}>2\frac{a_{1}}{a_{2}}N_{p},(2){\left(\frac{27a_{1}^{2}b_{1}}{8a_{2}^{2}b_{2}}\right)}^{2}=\frac{1}{27}{\left(\frac{1}{4}N_{s}^{2}-\frac{a_{1}}{2a_{2}}N_{p}\right)}^{3}$$

As for the fixed points *(y_s_^(2)^, y_p_^(2)^), (y_s_^(3)^,y_p_^(3)^)*, their stable condition is $y_{s}\in A\cap B$; in this situation, the parameters should satisfy the following conditions:

$$(1){\left(\frac{a_{1}}{b_{1}}\right)}^{2}+N_{s}^{2}<2\frac{a_{1}}{a_{2}}N_{p},(2){\left(\frac{27a_{1}^{2}b_{1}}{8a_{2}^{2}b_{2}}\right)}^{2}<\frac{1}{27}{\left(\frac{1}{4}N_{s}^{2}-\frac{a_{1}}{2a_{2}}N_{p}\right)}^{3}$$

where *A*, *B*, *T_1_* and *T_2_* are denoted by the following:

$$\begin{array}{l} A=\left[\frac{1}{2}\left(\frac{a_{1}}{b_{1}}+N_{s}-\sqrt{{\left(\frac{a_{1}}{b_{1}}\right)}^{2}+N_{s}^{2}-2\frac{a_{1}}{a_{2}}N_{p}}\right),\frac{1}{2}\left(\frac{a_{1}}{b_{1}}+N_{s}+\sqrt{{\left(\frac{a_{1}}{b_{1}}\right)}^{2}+N_{s}^{2}-2\frac{a_{1}}{a_{2}}N_{p}}\right)\right],\\ B=\left[-\frac{1}{2}\left(T_{1}+T_{2}-N_{c}\right)+\frac{\sqrt{3}}{2}i\left(T_{2}-T_{1}\right),T_{1}+T_{2}+\frac{N_{s}}{2}\right],\\ T_{1}=\sqrt[{3}]{-\frac{27}{8}{\left(\frac{a_{1}}{a_{2}}\right)}^{2}\frac{b_{1}}{b_{2}}+\sqrt{{\left(\frac{27a_{1}^{2}b_{1}}{8a_{2}^{2}b_{2}}\right)}^{2}+\frac{1}{27}{\left(\frac{a_{1}}{2a_{2}}N_{p}-\frac{1}{4}N_{s}^{2}\right)}^{3}}},\\ T_{2}=\sqrt[{3}]{-\frac{27}{8}{\left(\frac{a_{1}}{a_{2}}\right)}^{2}\frac{b_{1}}{b_{2}}-\sqrt{{\left(\frac{27a_{1}^{2}b_{1}}{8a_{2}^{2}b_{2}}\right)}^{2}+\frac{1}{27}{\left(\frac{a_{1}}{2a_{2}}N_{p}-\frac{1}{4}N_{s}^{2}\right)}^{3}}}. \end{array}$$

Theorem 2 If Δ=0, there are three nonzero fixed points in the natural gas supply system, where $y_{s}^{\left(2\right)}=y_{s}^{\left(3\right)}=\sqrt[{3}]{\frac{q}{2}}+\frac{2}{3}N_{s},y_{s}^{\left(1\right)}=-\sqrt[{3}]{4q}+\frac{2}{3}N_{s}$. If p = q = 0, the outcome will be three zero root. If the three fixed points are stable points, they should satisfy the condition of $y_{s}\in A\cap C$. Namely, the parameters should satisfy the following conditions:

$$\begin{array}{l} (1){\left(\frac{a_{1}}{b_{1}}\right)}^{2}+N_{s}^{2}>2\frac{a_{1}}{a_{2}}N_{p},(2){\left(\frac{27a_{1}^{2}b_{1}}{8a_{2}^{2}b_{2}}\right)}^{2}=\frac{1}{27}{\left(\frac{1}{4}N_{s}^{2}-\frac{a_{1}}{2a_{2}}N_{p}\right)}^{3}\\ C=\left(-\infty ,-2\sqrt[{3}]{{\left(\frac{a_{1}}{a_{2}}\right)}^{2}\frac{b_{1}}{b_{2}}}\right) \end{array}$$

Theorem 3 If Δ<0, there are three nonzero and unequal fixed points in the natural gas supply system. They must satisfy the condition of $y_{s}\in A\cap B$if they are stable fixed points. Namely, the parameters should satisfy the following conditions:

$$(1){\left(\frac{a_{1}}{b_{1}}\right)}^{2}+N_{s}^{2}<2\frac{a_{1}}{a_{2}}N_{p},(2){\left(\frac{27a_{1}^{2}b_{1}}{8a_{2}^{2}b_{2}}\right)}^{2}<\frac{1}{27}{\left(\frac{1}{4}N_{s}^{2}-\frac{a_{1}}{2a_{2}}N_{p}\right)}^{3}$$

### Simulation

#### Simulation of fixed points

**Fig. 1:**
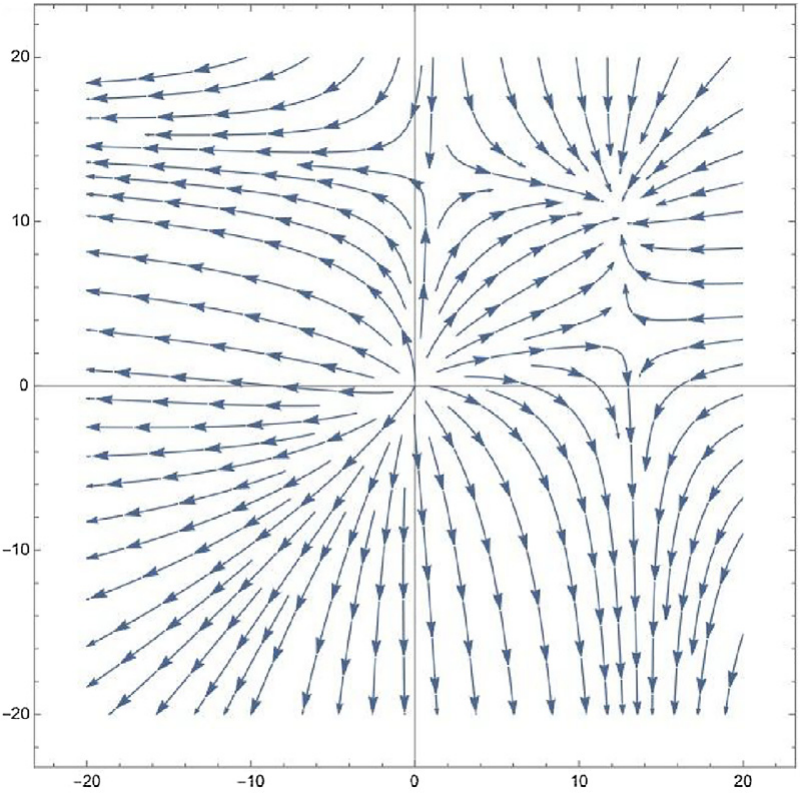
Phase diagram.

The gas supply information of Sino-German Ecopark in Qingdao, China, was taken as the data source, yielding *a_1_ = 0.25, a_2_ = 0.3, b_1_ = 0.6, b_2_ = 0.25, N_c_=N_m_ = 13.14.* Using Eq. (6), four fixed point coordinates were obtained: (12.941, 3.09055), (0.882218,12.9768), (12.4568,10.2126) and (0,0), complying well with the relationship demonstrated in Eq. (7). According to **Theorem 3**, (12.4568,10.2126) can be seen as a stable node. Next, simulations of the natural gas supply model were undertaken using the upper parameters of the Sino-German Ecopark data source. The phase diagram of the natural gas supply model was then plotted (where *x = y_s_, y = y_p_*), as shown in Fig. 1. From the latter, it can be seen that (0,0) is an unstable node, (12.941,3.09055) and (0.882218, 12.9768) are saddle points, and (12.4568,10.2126) is a stable node, which is in line with the above theoretical analysis.

The implications of Fig. 1 are as follows: (1) the maximum load of the natural gas supply from SNG and PNG is (12.4568, 12.9768) without manual intervention. This means that once users’ natural gas demand is over 25.4, they will face a shortage of gas and will need to replenish their supply in this circumstance. (2) Although the gas supply will be stable over a given period of time, it may become trapped in another supply branch at some point. For example, saddle points (12.941,3.09055) and (0.882218,12.9768) are stable in one direction but unstable in others.

#### Simulation with perturbation

The above simulation analysis was conducted under the condition that the system was in an ideal state and not subject to disturbance. The current section analyzes what could happen if the system is disturbed by Ref. [[2], [3], [4], [5]]. This involves, firstly, the natural gas supply being standardized with Np = Ns = 1.

An important research domain to handle disturbances/perturbations is perturbation observer based robust control [34] researched wind energy conversion systems under nonlinear perturbation. In wind energy conversion systems, the strong nonlinearities originated from the aerodynamics of the wind turbine, together with the generator parameter uncertainties and wind speed randomness. They aggregated above perturbation into a perturbation that is estimated online by a sliding-mode state and perturbation observer (SMSPO) [35] researched solar energy system. They aggregated the PV inverter nonlinearities and parameter uncertainties, unmodelled dynamics, stochastic fluctuation of atmospheric conditions, and external disturbances into a perturbation, which is then rapidly estimated online by a high-gain state and perturbation observer. In the end, they used PoFoPID control to handle various uncertainties and a great robustness can be achieved.

In [34] and [35], they mainly focused on monitoring and control the perturbation from environment and generator in process of producing energy. Different form above [34] and [35], we focus on the supply process of SNG and PNG. So, we just focus on the effect of disturbance on energy supply processes and not any control. In addition, in the process of SNG and PNG, the uncertainty of energy supply system is less and more stable, and the disturbance mainly comes from inside the system. SNG production is usually affected by stable disturbances ε1, while the PNG supply belongs to a pipeline supply that is relatively unstable and may be affected by a disturbance$-\varepsilon_{2}\cos y_{p}-\varepsilon_{3}\sin y_{p}$ in its supply process.

On the basis of Eq. (4), the equation with perturbation can be written as:

(9) $$\left\{\begin{array}{c} \frac{dy_{s}}{dt}=-a_{1}y_{p}+a_{2}(1-y_{s})y_{s}+\varepsilon_{1}\\ \frac{dy_{p}}{dt}=-b_{1}y_{s}+b_{2}(1-y_{p})y_{p}-\varepsilon_{2}\cos y_{p}-\varepsilon_{3}\sin y_{p} \end{array}\right)$$

If undertaking the second Taylor expansion for the disturbance$-\varepsilon_{2}\cos y_{p}-\varepsilon_{3}\sin y_{p}$, the following is obtained:

(10) $$\left\{\begin{array}{c} \frac{dy_{s}}{dt}=-a_{1}y_{p}+a_{2}(1-y_{s})y_{s}+\varepsilon_{1}\\ \frac{dy_{p}}{dt}=-b_{1}y_{s}+b_{2}(1-y_{p}-\varepsilon_{3})y_{p}-\varepsilon_{2}+\frac{y_{p}^{2}}{2}\varepsilon_{2} \end{array}\right)$$

To simplify the system, if *y_c_*=*y_s_*-(*a_2_*+1)/ (2*a_2_*) and *y_m_=-y_p,_* the mixed gas supply system with a disturbance (Eq. (10)) can be written as follows:

(11) $$\left\{\begin{array}{c} \frac{dy_{c}}{dt}=-a_{2}y_{c}^{2}-y_{c}+a_{1}y_{m}+\frac{a_{2}^{2}-1}{4a_{2}}+\varepsilon_{1}\\ \frac{dy_{m}}{dt}=b_{1}(y_{c}+\frac{a_{2}+1}{2a_{2}})+b_{2}\left(1-\varepsilon_{3}\right)y_{m}+(b_{2}-\frac{\varepsilon_{2}}{2})y_{m}^{2}+\varepsilon_{2} \end{array}\right)$$

Then, if *a_1_ = 1*, *b_1_ = 0.3*,$a_{2}\in [0.2,2]$,$\varepsilon_{3}=1+\frac{1}{b_{2}}$, and *y_c_=x*, it is possible to conduct a simulation of the above dynamic system. Since the disturbance of PNG is relatively stable and the amplitude of the disturbance is bounded, it can be assumed that the amplitude satisfies the condition of $\frac{\varepsilon_{2}}{2}(\varepsilon_{3}-1)=1$; thus, the dynamic equation can be written as follows:

$$\left\{\begin{array}{c} \frac{dy_{c}}{dt}=-a_{2}y_{c}^{2}-y_{c}+y_{m}+\frac{a_{2}^{2}-1}{4a_{2}}+\varepsilon_{1}\\ \frac{dy_{m}}{dt}=0\text{.3}y_{c}-y_{m}+\varepsilon_{2}+\frac{3a_{2}+3}{20a_{2}} \end{array}\right)$$

Following this, the force term of the above system was analyzed according to three situations, aiming to discern the relationship between robustness with disturbance and parameters by the maximum lyapunov exponent and bifurcation theory. The robustness of the system is analyzed from the two aspects of the change range of *a_2_* from stability to bifurcation and the magnitude of the maximum Lyapunov exponent with the change of *a_2_*. Generally speaking, the larger variation range of *a_2_* and the smaller the maximum Lyapunov exponent means the better robust.

$\text{Situation 1}:\varepsilon_{1}+\frac{a_{2}^{2}-1}{4a_{2}}=0,\varepsilon_{2}+\frac{3a_{2}+3}{20a_{2}}\neq 0\;\;\text{or}\;\varepsilon_{1}+\frac{a_{2}^{2}-1}{4a_{2}}\neq 0,\varepsilon_{2}+\frac{3a_{2}+3}{20a_{2}}=0.$

**Fig. 2:**
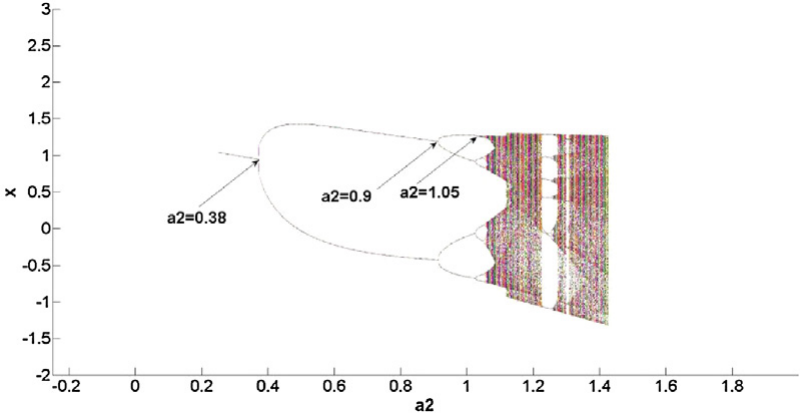
Bifurcation at situation 1.

**Fig. 3:**
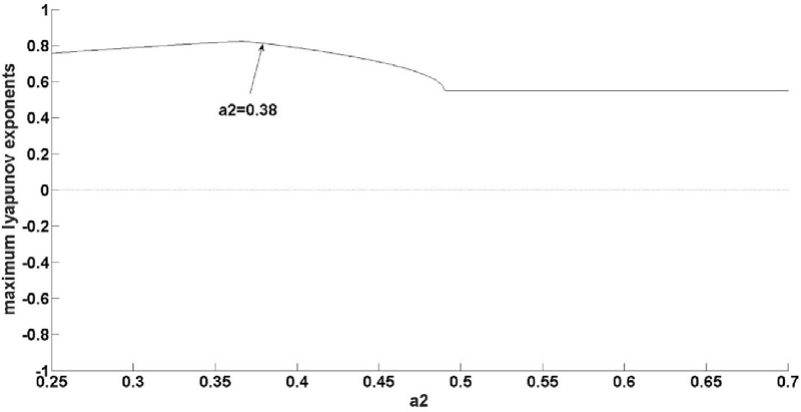
Maximum Lyapunov at situation 1.

For a better understanding of the above system dynamics, the robustness was analyzed when *a_2_* changes, as show in Fig. 2. From this, it can firstly be seen that the maximum Lyapunov exponent is more than 0 in Fig. 3, meaning that the natural gas supply system is a chaotic system. Secondly, the chaotic characteristics of the above system were analyzed.

As shown in Fig. 2, the robustness of the natural gas supply system varies with *a_2._* As evident in Fig. 3, when *a_2_*<0.38, the maximum Lyapunov exponent gradually increases, suggesting that the supply system evolves towards chaos with acceleration. In fact, in Fig. 2 it can be found that the natural gas system occurs bifurcation firstly at 0.38; namely, when *a_2_*<0.38, although the natural gas supply system is stable, it is sensitive to the change of system parameter. Therefore when *a_2_*<0.38, with the increase of *a_2_*, the robustness of the system becomes worse. Next, as demonstrated in Fig. 3, when 0.38<*a_2_*<0.9, the maximum Lyapunov exponent gradually decreases and, once *a_2_* reaches 0.52, remains constant. This suggests that the system evolves towards chaos with deceleration. In fact, in Fig. 2, it is found that natural gas supply system occurs bifurcation at 0.9. From stability to bifurcation, the range of change for *a_2_* is 0.52; thus, when 0.38<*a2*<0.9, the natural supply system is more robust than *a_2_*<0.38. When 0.9<*a_2_<*1.05, the maximum Lyapunov exponent is infinite, meaning that the system is extremely unstable. In fact, from Fig. 2 it is evident that when *a_2_*>1.05, the system falls into chaos, so when 0.9<*a_2_*<1.05, the robustness of natural supply system is poor. That is, the system is extremely sensitive to changes in parameter.

$\text{Situation 2}:\varepsilon_{1}+\frac{a_{2}^{2}-1}{4a_{2}}\neq 0,\varepsilon_{2}+\frac{3a_{2}+3}{20a_{2}}\neq 0$

As shown in Fig. 5, the maximum Lyapunov exponent is over 0, meaning that the natural gas supply system is a chaotic one. The chaotic characteristics of the latter system are now analyzed.

**Fig. 4:**
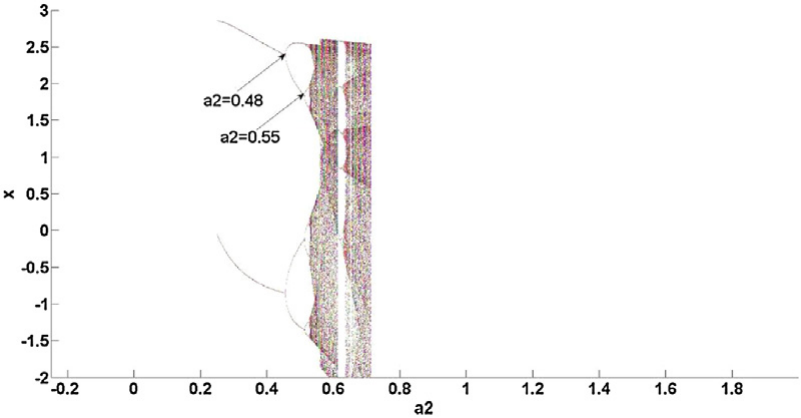
Bifurcation at situation 2.

**Fig. 5:**
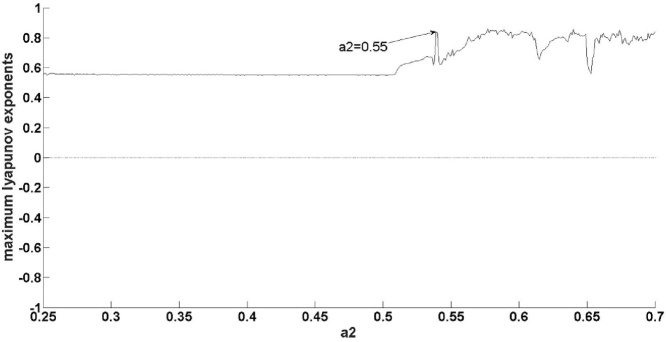
Maximum Lyapunov at situation 2.

Fig. 4 shows that the robustness of the natural gas supply system varies by *a_2._* In Fig. 5, when *a_2_*<0.48, it can be seen that the maximum Lyapunov exponent is stable, suggesting that the system evolves towards chaos with a uniform motion; this reflects the system’s superior robustness compared with situation 1: when *a_2_*<0.38, the maximum Lyapunov exponent gradually increases. In fact, in Fig. 4 it can also be found that the natural gas system occurs bifurcation firstly at 0.48, that means the system is more insensitive to the change of parameter compared to situation 1: *a_2_*<0.38.Therefore, when *a_2_*<0.48, the natural gas system is more robust. Next, Fig. 5 demonstrates that when 0.48<*a_2_*<0.55, the maximum Lyapunov exponent gradually increases, indicating that the system evolves towards instability with acceleration. In fact, in Fig. 4 it can be seen that the natural gas supply system descends int0 chaos at *a* = 0.55. Compared to condition 1, 0.38<*a_2_*<1.05, *a_2_* has a smaller range, that means the system is more sensitive to the change of parameter. Therefore the system is less robust.

$\text{Situation 3}:\varepsilon_{1}+\frac{a_{2}^{2}-1}{4a_{2}}=0,\varepsilon_{2}+\frac{3a_{2}+3}{20a_{2}}=0$

**Fig. 6:**
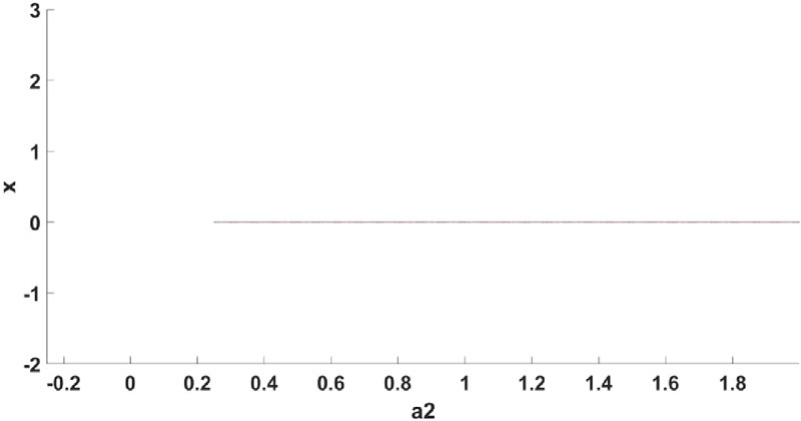
Bifurcation at situation 3.

As can be seen from Fig. 6, the natural gas supply is 0; this is inconsistent with the actual situation, making the model invalid under such conditions.

By analyzing the above three situations, it was found that if the amplitude of the disturbance satisfied $\varepsilon_{1}=\frac{\text{1-}a_{2}^{2}}{4a_{2}},\varepsilon_{2}=-\frac{3+3a_{2}}{20a_{2}}-1,\varepsilon_{3}=1-\frac{40a_{2}}{3+3a_{2}}$, the model could not explain the natural gas supply; this is, therefore, an area where the model needs further improvement. When the disturbance value satisfied$\varepsilon_{1}\neq \frac{\text{1-}a_{2}^{2}}{4a_{2}},\varepsilon_{2}\neq -\frac{3+3a_{2}}{20a_{2}}-1,\varepsilon_{3}\neq 1-\frac{40a_{2}}{3+3a_{2}}$, the system bifurcated firstly at *a_2_* = 0.48. Therefore, when *a_2_*<0.48, by the above analysis, the system can be said to have good robustness. The second bifurcation point occurs when *a_2_* = 0.55, whereby the system falls into a chaotic state after *a_2_* reaches 0.55. Therefore, when 0.48<*a_2_*<0.55, with the increase of *a_2_*, the maximum Lyapunov exponent increases gradually, the system can easily descend into a chaotic state, meaning that the system is in a weak-robust state in intervals of (0.48, 0.55). Therefore, it is necessary to control *a_2_*<0.48 in order to ensure the system has better robustness. When the perturbation value does not belong to the above two situations, the system bifurcation point the first time around is *a_2_* = 0.38, by the above analysis, indicating that the system has bad robustness when *a_2_*<0.38. The second bifurcation point occurs at *a_2_* = 0.9, and the system falls into a chaotic state after 1.05, meaning that it is easy for the system to fall into the latter state after *a_2_*>0.9; the system can thus be said to be in a worst robustness state in intervals of (0.9, 1.05). It is, therefore, necessary to control 0.38<*a_2_*<0.9, in this range, the system is in a good robust state.

The above analysis of the sensitivity of natural gas supply system to parameter under different disturbances gives the robustness of the system. Based on the above analysis, and using the characteristics of randomness, ergodicity and regularity in chaotic motion, the optimal parameter design of the natural gas supply system was found, which can make this system obtain better robustness.

## Conclusion

This work studied the stability of two kinds of energy in the supply process and the robustness of the supply system under different disturbances. Through establishment of the dynamic system model and the numerical simulation of the two energy supply processes, this paper found three essential questions: (1) the stability of fixed point in natural gas supply system is determined by system parameters. (2) The output values of two kinds of energy sources in the natural gas supply system are constant in the state of stable fixed point, and the output values of two kinds of energy sources are uncertain in the state of unstable fixed point. (3) Natural gas supply system is a fragile system, under parameter perturbation, the ability of the system to maintain original performance is affected by disturbance type of internal and external disturbances.

The existing literatures on the reliability of energy supply process were mainly data-driven and results-oriented. They had their own advantages, but they were unable to reveal the internal evolution mechanism of the natural gas supply process and provided guidance for improving the robustness of the natural gas supply system.

Different from the existing methods, this paper can gain evolution law of the natural gas supply, and provide guidance for improving the robustness of the natural gas supply system. Two practical significance of this paper: (1) by analyzing the stability of fixed points in the gas supply system, the basis for judging stability was yielded. It is hoped that this can, in turn, help gas suppliers judge the stability of the natural gas supply. In addition, this may also help gas suppliers to judge whether the supply of natural gas is adequate and when it needs to be replenished, in order to avoid gas shortages. (2) By changing the coefficient *a_2_*, the critical point was found at which the natural gas supply system would encounter bifurcation under different perturbations. This finding links different disturbances to system robustness and may help enterprises to optimize the design of their system parameters under different perturbations, in order to prevent the gas supply system from turbulence even chaos. Furthermore, the findings of the current study discovery may help gas suppliers to uncover the causes of bifurcation in the gas supply system, which may include the price of coal, or the cost of environmental pollution caused by the conversion of coal into SNG, among other factors.

This paper focuses on the dynamic stability of energy supply processes.The stability condition in energy supply process is given.In the future research, this paper focuses on chaos control.That is, when the stability of energy supply process changes and chaos occurs, for example, Fig. 2, Fig. 4, how to carry out effective control to ensure the system to restore the stable supply state.

## Funding

This work was supported by National Natural Science Foundation of China: Research on Nodes Configuration of Variant Design Based on Coupling Evolution (51465020).
